# Zika virus disrupts the barrier structure and Absorption/Secretion functions of the epididymis in mice

**DOI:** 10.1371/journal.pntd.0009211

**Published:** 2021-03-05

**Authors:** Ziyang Sheng, Na Gao, Dongying Fan, Na Wu, Yingying Zhang, Daishu Han, Yun Zhang, Weilong Tan, Peigang Wang, Jing An

**Affiliations:** 1 Department of Microbiology, School of Basic Medical Sciences, Capital Medical University, Beijing, China; 2 Laboratory Animal Center, Capital Medical University, Beijing, China; 3 Institute of Basic Medical Sciences, School of Basic Medicine, Chinese Academy of Medical Sciences, Peking Union Medical College, Beijing, China; 4 Huadong Research Institute for Medicine and Biotechnics, Nanjing, Jiangsu, China; 5 Center of Epilepsy, Beijing Institute for Brain Disorders, Beijing, China; WRAIR, UNITED STATES

## Abstract

Several studies have demonstrated that Zika virus (ZIKV) damages testis and leads to infertility in mice; however, the infection in the epididymis, another important organ of male reproductive health, has gained less attention. Previously, we detected lesions in the epididymis in interferon type I and II receptor knockout male mice during ZIKV infection. Herein, the pathogenesis of ZIKV in the epididymis was further assessed in the infected mice after footpad inoculation. ZIKV efficiently replicated in the epididymis, and principal cells were susceptible to ZIKV. ZIKV infection disrupted the histomorphology of the epididymis, and the effects were characterized by a decrease in the thickness of the epithelial layer and an increase in the luminal diameter, especially at the proximal end. Significant inflammatory cell infiltration was observed in the epididymis accompanied by an increase in the levels of interleukin (IL)-6 and IL-28. The expression of tight junction proteins was downregulated and associated with disordered arrangement of the junctions. Importantly, the expression levels of aquaporin 1 and lipocalin 8, indicators of the absorption and secretion functions of the epididymis, were markedly reduced, and the proteins were redistributed. These events synergistically altered the microenvironment for sperm maturation, disturbed sperm transport downstream, and may impact male reproductive health. Overall, these results provide new insights into the pathogenesis of the male reproductive damage caused by ZIKV infection and the possible contribution of epididymal injury into this process. Therefore, male fertility of the population in areas of ZIKV epidemic requires additional attention.

## Author summary

Unlike other mosquito-transmitted flaviviruses, ZIKV can persistently replicate in the male reproductive system and is sexually transmitted. ZIKV infection was reported to damage testis. However, ZIKV-induced epididymal injury was not investigated in detail. Clinically, epididymitis is closely associated with male infertility. In this study, a mouse model was used to demonstrate that ZIKV causes histomorphological and functional changes in the epididymis, which may alter the microenvironment of sperm maturation and movement and finally lead to male infertility. Therefore, long-term investigation of male reproductive health may be needed in the areas of ZIKV epidemic.

## Introduction

Zika virus (ZIKV) is a mosquito-borne flavivirus that has rapidly spread worldwide in recent years. Unlike other flaviviruses, ZIKV can persistently replicate in the male reproductive system and has been shown to be the only flavivirus that is sexually transmitted [1–4]. Our previous studies and the work of other authors demonstrated that ZIKV infection damages testis and leads to infertility in mice [5–10]. We also detected apparent lesions of the epididymis, including hyperemia and swelling [7]. Clinically, epididymitis is commonly attributed to an ascending bacterial infection via the vas deferens downstream and has significant impact on sperm quality [11–13]. Persistent oligo- or azoospermia or reduced sperm motility were noted in patients with epididymitis after the recovery [14–17]. Moreover, the incidence of epididymitis is considerably higher than that of orchitis in humans [18]. Therefore, the significance of the epididymis in male fertility suggests that the pathogenesis of ZIKV infection in the epididymis should be investigated. The epididymis has been reported as an important target for ZIKV *in vivo* and was damaged during infection [9,10]. However, more details are needed and the pathogenesis still lacks.

The epididymis contains a highly convoluted duct that connects testis and vas deferens. The epididymis can be divided into distinct anatomical segments: the bulbous initial segment (IS) with the caput at the proximal end, elongated corpus, and inflated cauda at the distal end [19–21]; each segment has distinct morphology and function. Histology of the epithelium varies between the segments from proximal to distal. The epididymal duct at IS is characterized by a small tubule diameter and irregular height of the epithelium with true cilia. The epithelium of the caput tubule is comprised of tall, columnar epithelial cells of regular height with dense microvilli lining the lumen. From the corpus to the cauda, the tubule diameter increases, and epithelial cells gradually become thinner. The tubule has a higher density of spermatozoa in the cauda than that in the upstream regions [21]. The epithelial cells in the epididymis are referred to as EEC and more than 80% are principal cells [22,23].

The epididymis plays critical roles in sperm maturation, transport, storage, and protection by providing a suitable microenvironment [24–26]. The distinct segments are closely associated with the epididymal functions [27]. Male gametes leave the testis in a functionally immature status. They acquire the motility and fertilization capacity downstream during transportation and are finally stored in the cauda [28]. The proximal end, especially IS, has absorptive and secretory functions that provide nutrition, and the distal end has higher absorbent ability to facilitate sperm movement [20]. Various proteins are secreted by the epididymis, including lipocalins (Lcns) and aquaporins (AQPs) that are important for the maintenance of the epididymal microenvironment for the sperms. Lcns are major transport proteins associated with sperm maturation [29–32]. Lcn5 and Lcn8, the rodent epididymal Lcns, bind retinoic acid, which is a vital regulatory factor in the male reproductive tract and facilitate the maturation of sperms [33]. AQPs are known as plasma membrane water-transporting proteins and are involved in the modulation of the water transport through the membrane of epididymal epithelium [34]. AQP1 is predominantly expressed in the epididymal epithelial cells and plays an important role in the secretion/reabsorption dynamics of luminal fluid during sperm transport and maturation [35]. Lcns and AQPs contribute to the control of the fluid movement and modulate the luminal environment of the epididymis [36,37].

During the transport and maturation of the sperm, the blood-epididymis barrier (BEB) is important for the regulation of the local microenvironment to sequester autoantigenic spermatozoa from the immune system and guarantee proper development of the germ cells into fully functional sperms [28,38,39]. Epithelial tight junctions of the epididymis contribute to the formation of the BEB of maximum competence [33]. Several tight junction-associated proteins are involved in the formation of the BEB. Among the various epithelial cell contacts, zonula occludens-1 (ZO-1) has been indicated to be the most highly distributed in the epididymis in most mammals [39,40]. ZO-1 is a peripheral membrane protein involved in tight junctions between adjacent principal cells, which are crucial for the BEB [41,42]. A decrease in the expression of ZO-1 indicates an impairment of the barrier [43]. Claudin-1 (Cldn-1) is important for survival in mice, and a lack of Cldn-1 cannot be compensated by other tight junction proteins [44,45]. Cldn-1 is localized in epididymal tight junctions in all epididymal regions [46]. Therefore, BEB contributes to the immunological privileges of the epididymis. BEB integrity, normal secretion, and resorption by the lining epithelial cells play important roles in the maintenance of the inner tubular microenvironment in the epididymis.

Experimental evidences of mice showed the epididymis is more susceptible to inflammation and immune reactions than the testis [18,47,48]. The epididymal duct is surrounded by a peritubular layer of smooth muscle cells. An interstitial tissue stroma fills the space containing vasculature and lymphatics. Macrophages are the major epididymal immune cells that are predominantly detected in the interstitial and peritubular regions [16]. Dendritic cells form a dense network in the basal region of the epithelium and extend apically between the epithelial cells. These cells appear to be particularly active at the proximal caput [49].

Wild type mice are not natural hosts for ZIKV and cannot support continuous virus replication. Therefore, in our previous study, to mimic the natural infection way, the IFN I and II receptors knockout (AG6) mice were inoculated with ZIKV in footpads to investigate the effect of ZIKV on testis. To comprehensively determine the effect of ZIKV on male reproductive health, we selected this mouse model to investigate the pathogenesis of ZIKV in the epididymis. Histology was analyzed in the segments. Furthermore, the changes in the epididymal barrier structure and levels of proteins involved in the secretion and absorption were determined after the infection to evaluate the changes in the epididymal functionality. Our data may provide new insights into the pathogenesis of the male reproductive damage caused by ZIKV infection and the possible contribution of epididymal injury into this process.

## Materials and methods

### Ethics statement

All animal experimental procedures were approved by the Experimental Animal Welfare and Ethics Committee of Capital Medical University, Beijing, China (Approval number: AEEI-2015-048). All efforts were made to minimize animal suffering.

### Cells and virus

Aedes albopictus (C6/36) cells were maintained at 28°C in RPMI 1640 (Gibco, USA) with 10% fetal bovine serum (FBS, PAN, Germany). ZIKV (strain CAS-ZK01) was propagated in C6/36 cells and stored at -80°C until use. Stock titers were determined by plaque assay in Vero cells.

### Mouse experiments

AG6 mice were kept in specific-pathogen-free environments. 8 week-old male mice were challenged with 10^5^ plaque-forming units (PFU) ZIKV into bilateral footpads. Mice were euthanized after isoflurane-induced deep anesthesia by cervical dislocation at 2, 5 and 8 days post infection (dpi). Testes and epididymides were harvested after dissection. Uninfected mice served as control. There were 3 mice for transcriptase analysis and 5 mice for the other experiments including morphological, functional analysis and viral test experiments.

### Measurement of viral loads

The entire epididymis of ZIKV-infected or control mice were homogenated in Trizol (Transgen, China). After total RNA was regularly extracted, real-time qPCR (RT-qPCR) was used to determine the copies of ZIKV mRNA on an ABI 7500 Real Time PCR System (Applied Biosystems, USA). A pair of primers was designed to detect ZIKV mRNA (forward: 5′-TCAGACTGCGACAGTTCGAGT-3′; reverse: 5′-GCATATTGACAATCCGGAAT-3′). Quantification of the copies of ZIKV mRNA was determined by standard curve method. The method was applied in our previous publication [7].

### Histological examination

Epididymis was fixed in Modified Davidson’s Fluid and embedded in paraffin. The slices were prepared according to standard method.

For morphological analysis, the sections were stained with Hematoxylin and Eosin (HE) and observed under bright-field microscopy. Measurement of tubular diameters and epithelial thickness of epididymal duct were performed using ImageJ. Measurements of epithelial thickness and lumen diameter were performed according to previous reports [50,51]. Briefly, 10 samples were used to analyze epididymal tubule diameter and epithelial thickness in each group (Bilateral epididymides of 5 mice). Five images in each segments and five cross sections of epididymal duct in each image were analyzed in each sample. We selected the epididymal duct, whose length of the major axis was at most 1.5 times that of the minor axis. Each tubule diameter and epithelial thickness were measured at the average of the maximal and minimal distance. The average of five diameters and thickness were calculated as the representative data for one sample respectively.

For ultrastructure observation, epididymides were fixed in glutaraldehyde. The ultrathin sections were prepared according to standard method and observed under transmission electron microscope (H-7500, HITACHI). Epididymides of three mice were observed in uninfected control and ZIKV-infected at 8 dpi groups, respectively. The method was described in our previous publication [7].

### Immunohistochemistry staining

For detecting immunocytes infiltration and functional protein expression, the sections were subjected to immunohistochemistry staining (IHC). Briefly, the sections were deparaffinized in xylene and rehydrated through descending graded alcohols to phosphate-buffered saline (PBS, pH 7.4). Endogenous peroxidase activity were blocked with 3% hydrogen peroxide for 10 minutes. Then the slides were incubated with 1% bovine serum albumin (in PBS) to block nonspecific antibody binding sites. For antigen retrieval, slides were boiled in 10 mmol/L sodium citrate buffer (pH 6.0; ZLI-9064, Zhongshan Golden Bridge Bio Co., Ltd., China) 3 times for 10, 5, and 5 minutes, respectively, in a microwave oven (800 W). Afterwards, the specimens were incubated with anti-CD45 (1:200, Thermo Fisher, 33–9100), anti-CD11c (1:200; Thermo Fisher, 33–9100), anti-F4/80 (1:200; Abcam, ab10598), anti-Lcn8 (1:500; Bioss, bs-18196R) or anti-AQP1(1:500; Abcam, ab65837) at 4 °C overnight, and followed by incubation with horseradish peroxidase (HRP)-conjugated goat anti-mouse or rabbit IgG. Finally, after developing color and counterstaining with hematoxylin, slides were examined under a light microscope. The detailed method was applied the previous publication [52].

### Immunofluorescence staining

Frozen sections of the epididymis from AG6 mice were prepared and subjected to immunofluorescence assay (IFA).

For detecting the distribution of viral antigens, the sections were incubated with mouse anti-ZIKV polyclonal serum produced in our lab (1:500) at 4 °C overnight, followed by incubation with Alexa Fluor 488 goat anti-mouse IgG (H+L) (1:400; Life technologies, A11001).

For analyzing the changes of tight junctions in epididymides, the sections were incubated with anti-mouse ZO-1(1:200; Thermo Fisher, 33–9100) or Cldn-1 (1:200; Abcam, ab10598) at 4 °C overnight and followed by incubation with Alexa Fluor 488 goat anti-mouse IgG or Alexa Fluor 594 donkey anti-rabbit IgG (H+L) (1:400; Life technologies, A21207). All images were captured with a laser confocal scanning microscopy (Leica TCS SP5).

### Infection experiments with Primary simian cells *in vitro*

A healthy two-year-old male African green monkey was euthanatized by bloodletting after deep anesthesia. Testis and epididymis were harvested. Primary simian cells, including Sertoli cells (SC) and Leydig cells (LC) in testis, epididymal epithelial cells (EEC) in epididymis were isolated.

For isolating LC, tunica-free testes were incubated with 0.25 mg/mL collagenase type I (Sigma, US) at 32 °C for 10 min. The suspensions were then filtered and the interstitial cells were cultured in F12/DMEM complete medium (Sigma, US) for 15 min. Then the non-adherent cells were resuspended by F12/DMEM medium and cultured at 34 °C. After 24 h, LC were detached by treatment with 0.125% trypsin for 5 min. For isolating SC, the seminiferous tubules were incubated with collagenase type I at 32 °C for 15 min and followed by further incubation with 1 mg/mL hyaluronidase (Sigma, US) at 32 °C for 10 min to separate germ cells and SC.

For isolating EEC, epididymides were incubated with 10 mg/ml collagenase type IV (Sigma) in F12/DMEM medium for 30 min. The epididymal tubules were filtered and treated with 0.5 mg/ml hyaluronidase (Sigma) for 15 min, followed by incubation with 1.0 mg/ml hyaluronidase for 30 min. The suspensions were filtered and EEC were cultured in F12/ DMEM media at 32 °C.

Primary simian reproductive cells were infected with ZIKV at a multiplicity of infection (MOI) of 1 for 1 h. Viral loads in supernatant and viral antigen within cells were detected by real-time PCR or IFA after infection. Three independent experiments were performed for each time point.

### Transcriptome analysis

RNA sequencing analysis of epididymides infected with ZIKV at 5 dpi or without infection was commercially conducted (Kangchen Biotech, China). Three individuals were in each group. In brief, after extraction of total RNA, mRNA was isolated by magnetic beads with Oligo (dT). The mRNA was fragmented and further enriched and purified by KAPA Stranded RNA-Seq Library Prep Kit (Illumina) to create cDNA libraries. Real-Time PCR System was used to quantify and qualify the sample libraries. Finally, the cDNA libraries were sequenced using HiSeq 2000 Sequencing System (Illumina, Inc., USA). Gene expression levels and the transcription levels (FPKM value) were analyzed by Cufflinks 2 software (v2.1.1) to screen differentially expressed genes between groups. To reflect the effect of ZIKV on epididymal Absorption/Secretion functions, the gene clusters of AQPs and Lcns were analyzed.

### Cytokines protein microarray

Cytokines in epididymides and testes at different time points were commercially determined by Quantibody Mouse Cytokine Array 1 (RayBiotech Co., China). Three individuals were in each group. In brief, antibody array was immobilized to a glass slide. The immobilized protein was incubated with homogenated samples. Then a second biotin-labeled detection antibody cocktail was added. After incubated with dye-conjugated streptavidin, the array was scanned using the Axon GenePix 4000B (Silicon Valley, CA) and data was collected using the GenePix Pro software. Levels of cytokines were displayed by signal intensity.

### Statistical analysis

GraphPad Prism 7.0 was used for all analyses. The analyses were conducted using ANOVA with a multiple comparison test or t test. p-values < 0.05 were considered statistical significance.

## Results

### Epididymal epithelial cells efficiently support ZIKV replication

**Fig 1:**
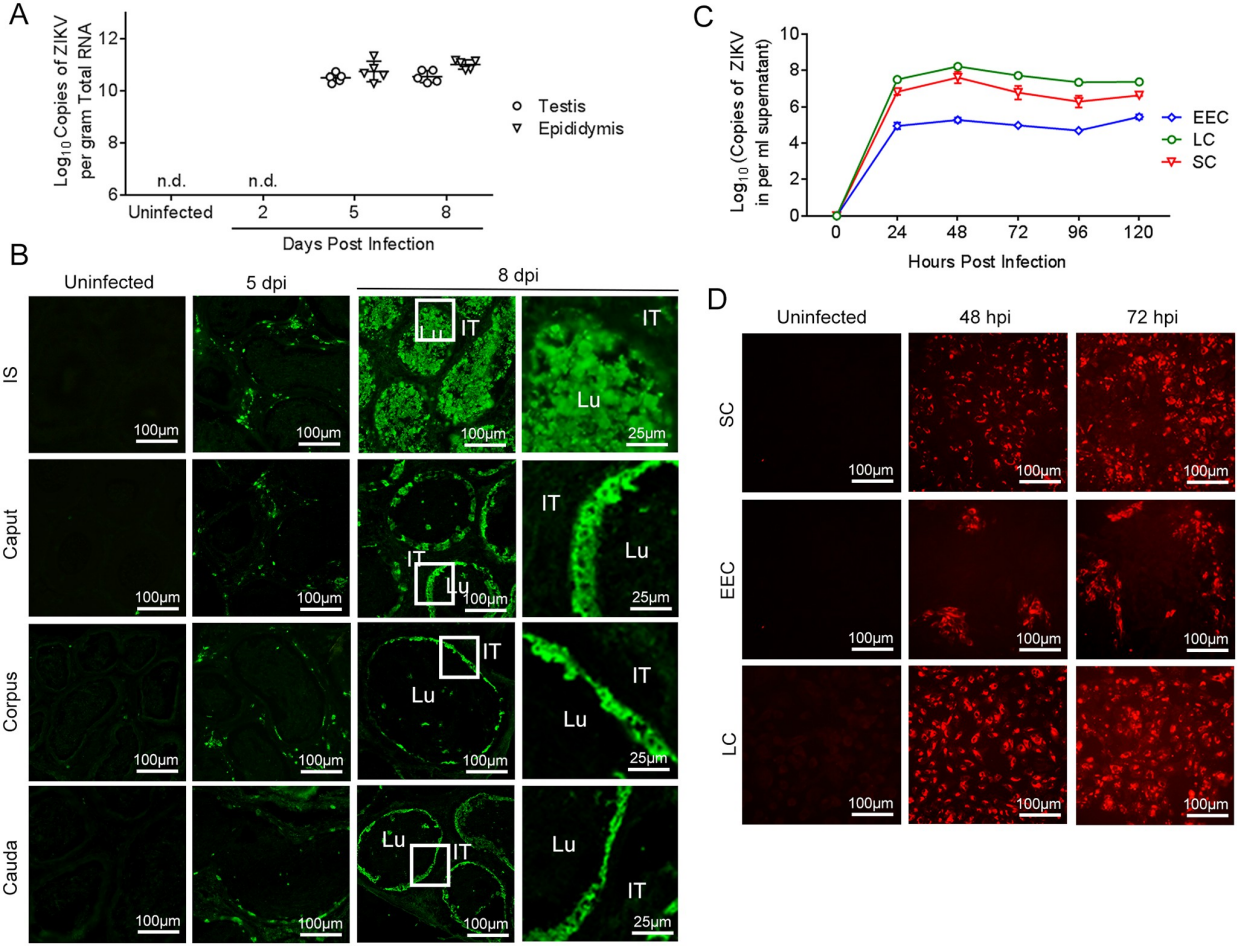
ZIKV replication in the mouse epididymis and simian primary cells. Eight-week-old male AG6 mice were inoculated with ZIKV at 10^5^ plaque-forming units (PFU) in bilateral footpads. (A) Viral loads in the testis and epididymis at 2, 5, and 8 days post infection (dpi) were detected by RT-qPCR. n = 5. Each data point represents an individual mouse. (B) Distribution of ZIKV antigens in various epididymal segments in AG6 mice at 2, 5, and 8 dpi was determined by immunofluorescent assay (IFA). IT, intertubular space; Lu, lumen. Primary Seroli cells (SC), epididymal epithelial cells (EEC) and Leydig cells (LC) were isolated from a two-year-old male African green monkey. Simian primary cells were infected with ZIKV at multiplicity of infection (MOI) = 1. Supernatant was harvested every 24 hours until 120 hours. (C) Viral proliferation kinetics in simian SC, EEC and LC were determined by RT-qPCR. Three independent experiments were repeated. (D) Distribution of ZIKV antigens in simian SC, EEC and LC at 48 and 72 hours post infection (hpi) was detected by IFA.

To determine the susceptibility of the epididymis to ZIKV, viral loads in the epididymis were analyzed by RT-qPCR and compared to those in the testis. At 2 dpi, ZIKV nucleic acids were not detected in either organ. However, at 5 dpi, high level of viral RNA was detected in the epididymis (10.55 ± 0.23 log_10_ copies/g tissue), similar to that in the testes (10.50 ± 0.18 log_10_ copies/g tissue). At 8 dpi, viral loads in both organs were slightly increased (11.01 ± 0.18 log_10_ copies/g in the epididymis and 10.75 ± 0.39 log_10_ copies/g in the testis)(Fig 1A). These results indicate that the epididymis was susceptible to ZIKV.

Viral distribution in the epididymis of infected AG6 mice was determined by IFA. Consistently, viral antigens were undetectable at 2 dpi but were sparsely distributed in the interstitial tissue around the ductus at 5 dpi; the distribution was similar in all regions. Notably, ZIKV antigens were aggregated at 8 dpi. The antigens were predominantly distributed in EEC in the caput and distal segments and were associated with abundant ZIKV antigens in the lumens of IS; the level of the antigens was gradually decreased in the downstream segments (Fig 1B). This result indicated that blood dissemination of ZIKV might be preferential and is followed by testicular exudation. EEC supports ZIKV replication.

To further verify the tropism of ZIKV to epididymis, simian primary EEC were isolated and infected with ZIKV *in vitro*. Meanwhile, testicular SC and LC were infected as positive controls. As shown in Fig 1C, EEC, SC and LC all efficiently supported ZIKV replication. Viral loads in cell supernatant peaked at 24 hours post infection (hpi) and maintained a stable level for up to 120 hpi. The distribution of viral antigens was further determined by IFA. ZIKV-positive EEC distributed in clusters at 48 and 72 hpi, which was significantly different from SC and LC, in which large amount of ZIKV antigens showed dispersed distribution (Fig 1D). These results further indicated the susceptibility of EEC to ZIKV infection.

### ZIKV infection severely disrupts the histological structure of epithelial layer in the epididymis

**Fig 2:**
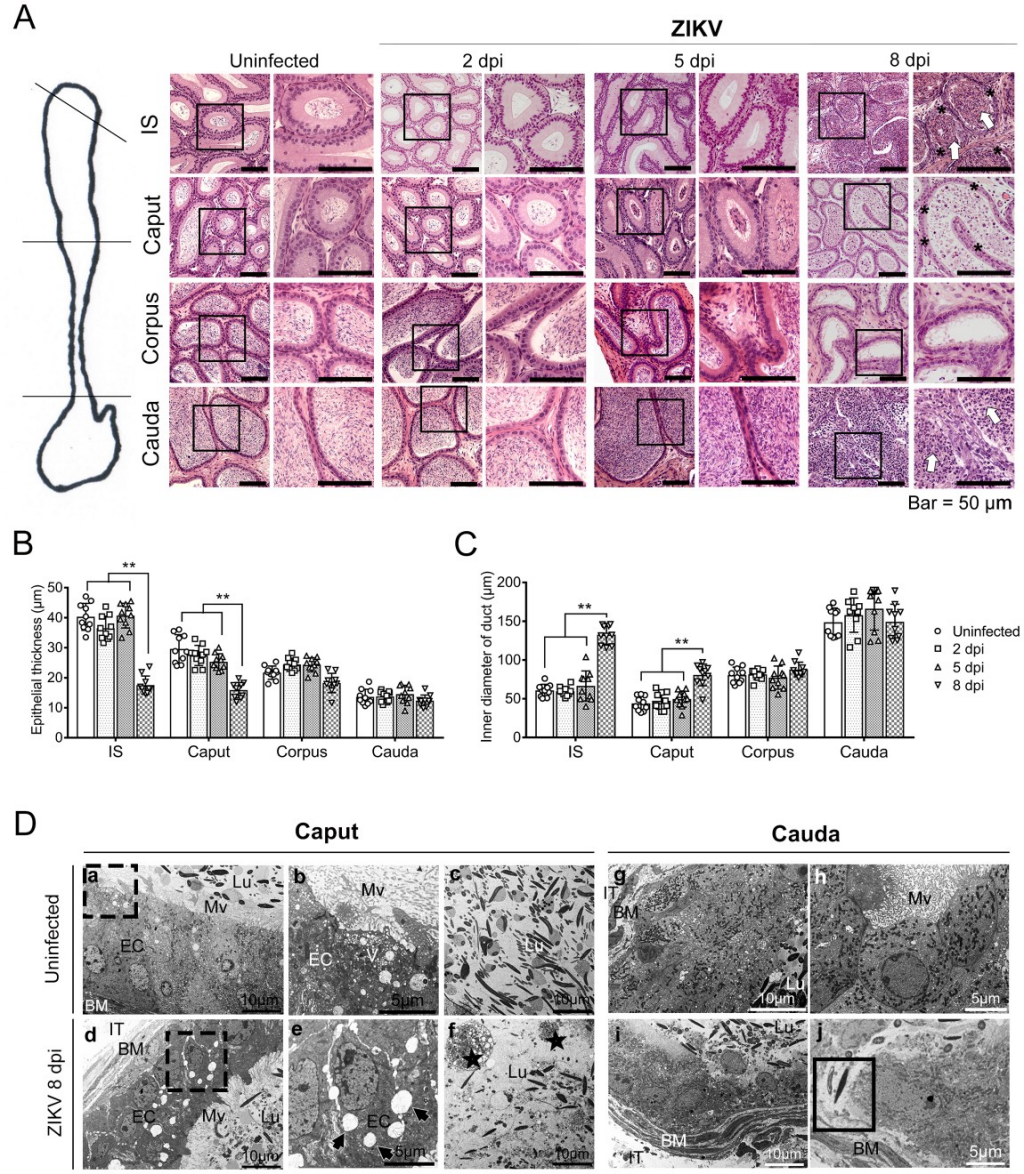
Histopathological changes in the epididymis after ZIKV infection. Eight-week-old male AG6 mice were inoculated with 10^5^ plaque-forming units (PFU) of CAS-ZK01 strain of ZIKV in bilateral footpads. Epididymis was collected at 2, 5, and 8 days post infection (dpi). Uninfected mice were used as a control. n = 5. (A) Histology of the epididymal segments was analyzed by HE staining. The atrophied epithelium was indicated by black asterisks. Blockage of cell debris in lumen was indicated by white arrows. The changes in epithelial thickness (B) and luminal diameter (C) of the segments at indicated time points were measured. Average epithelial thickness and luminal diameter in bilateral epididymis of 5 mice in each group were assessed. n = 10. *, P < 0.05 and **, P < 0.01, according to t test. (D) Ultrastructure of the caput (a-f) and cauda (g-j) with or without ZIKV infection was observed using a transmission electron microscope (TEM). Unilateral epididymis from 3 mice in each group was used. After ZIKV infection, epithelial thickness is considerably decreased, microvilli become sparse, and vacuoles (indicated by black arrows) are detected between the cells (a, b, d, e). The foam macrophages (indicated by black stars) are accumulated, and the density of the sperms in the lumen is decreased (c, f). In the cauda, epithelial cell layer is disrupted after the infection (g, h, i). Sperm directly contacts the basement membrane of the tubules (indicated by rectangle, j). IT, intertubular space; BM, basement membrane; Mv, microvilli; Lu, lumen; EC, epithelial cells; V, vesicles.

The effect of ZIKV infection on the epididymis was observed at 2, 5, and 8 dpi. In uninfected mice, epididymis had a thicker epididymal epithelium in the caput, and the thickness was progressively decreased along the tubule becoming the thinnest in the cauda. The lumen of each segment was filled with abundant sperms. After ZIKV infection, no obvious pathohistological changes were observed at 2 dpi. However, at 5 dpi, the epithelial cells of the epididymis were disorganized, and necrotic tissue and cells were detected in the lumen. Histology was significantly changed at 8 dpi (Fig 2A). The proximal epithelium was disorganized and its thickness was decreased (35.13 ± 6.03 μm in IS and 31.80 ± 5.10 μm in caput); these values were significantly lower than those in the control [80.63 ± 8.974 μm (p < 0.01) and 59.20 ± 9.49 μm (p < 0.01), respectively]. However, the thickness of the epithelium in distal ductus was decreased only slightly (Fig 2B). Moreover, ductus diameter in each region was also measured. After ZIKV infection, at 8 dpi, the proximal lumen was enlarged with a diameter of 265.31 ± 24.29 μm in IS and 161.44 ± 28.07 μm in the caput, respectively; these values were significantly different from the corresponding values in the control [121.42 ± 15.94 μm (p < 0.01) and 88.05 ± 18.62 μm (p < 0.01), respectively]; however, luminal diameter of the distal ductus was not affected (Fig 2C). Notably, necrotic cells were clearly observed in the tubular lumen in all segments at 8 dpi, especially in the proximal region, indicating that the transport of the sperms was obstructed. Numerous dense leukocytes were distributed in the interstitial tissue in all segments (Fig 2A).

Transmission electron microscopy (TEM) displayed that uninfected EECs in the caput were tightly lined in a regular arrangement (Fig 2D-a). A large number of vesicles filled the cytoplasm indicating strong secretory capacity of epididymal epithelial cells. Microvilli densely covered the apical membrane (Fig 2D-b). Abundant sperm heads filled the lumen (Fig 2D-c). After ZIKV infection, the EEC thickness was considerably decreased, microvilli were sparse, and vacuoles were observed between the cells in the epithelium (Fig 2D-d and 2D-e). The foamy macrophages were accumulated, and the density of the sperm in the lumen was decreased (Fig 2D-f). EEC in uninfected cauda had numerous vesicles inside and dense microvilli outside the cells, similar to those in the caput (Fig 2D-g and 2D-h). However, at 8 dpi, the number of microvilli was decreased, and EECs were detached in the cauda. Notably, sperms were in direct contact with the basement membrane in some areas of the tubules (Fig 2D-i and 2D-j). These results suggested that the integrity of the epithelial layer in all epididymal segments was severely disrupted, especially in the proximal ends.

### Immunological injury plays an important role in the epididymal lesions during ZIKV infection

**Fig 3:**
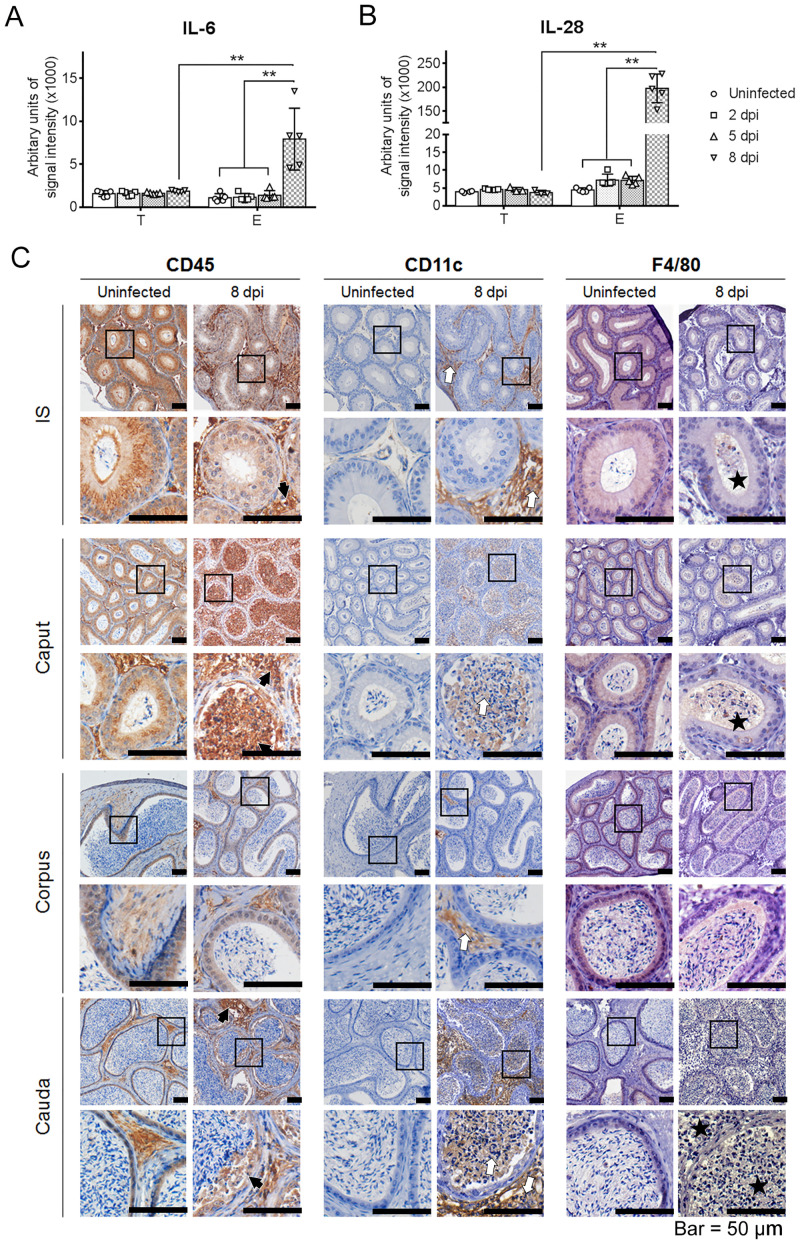
Inflammation in the epididymis after ZIKV infection. Eight-week-old male AG6 mice were inoculated with 10^5^ plaque-forming units (PFU) of the CAS-ZK01 strain of ZIKV in bilateral footpads. IL-6 (A) and IL-28 (B) in the epididymis and testis at 2, 5, and 8 dpi were detected by protein microarray. Each data point represents the results obtained in an individual mouse. n = 5. **, P < 0.01, according to the t test. (C) The types of immunocytes were determined by immunohistochemistry staining (IHC). CD45, CD11c, and F4/80 staining correspond to pan-leukocytes, myeloid dendritic cells, and macrophages marker, respectively. Changes in the expression and distribution were detected in the epididymis at 8 dpi after ZIKV infection (CD45 indicated by black arrows, CD11c indicated by white arrows, F4/80 indicated by black stars).

Cytokines were determined by protein array to characterize the profile. At 8 dpi, the levels of 18 tested cytokines were not significantly changed in the testis (S1 Fig). In contrast, IL-6 and IL-28 levels were considerably increased in the epididymis compared to those in the testes (Fig 3A and 3B) indicating severe inflammatory injury in the epididymis.

The types of infiltrating immunocytes in the epididymis were analyzed by IHC. The expression of CD45 (pan-leukocyte marker), CD11c (myeloid dendritic cells marker) and F4/80 (macrophage marker) were detected in the epididymis. As shown in Fig 3C, in uninfected epididymis, cells expressing CD45 were commonly detected in the epithelial layer and interstitial tissue in all segments. At 8 dpi, CD45 staining in the epithelial layer was significantly decreased and that in the interstitial tissue was substantially increased. Notably, numerous CD45^+^ cells filled the proximal lumen indicating severe inflammatory cell infiltration (Fig 3C). The expression of CD11c in uninfected epididymis was barely detectable in all segments. However, at 8 dpi, CD11c expression in the interstitial tissue in all segments was considerably increased and was accompanied by the presence of numerous CD11c^+^ cells in the lumen in the caput and cauda. Normally, F4/80 was predominantly distributed in the epithelial layer and barely detected in the interstitial tissue. After ZIKV infection, the expression of F4/80 within the epithelial layer was considerably decreased and that within the lumens was substantially increased; the expression of the antigens was increased downstream along the tubule. Thus, the pathogenesis indicated the inflammation contributed to ZIKV-induced epididymal lesion.

### Tight junctions in the epididymis are disrupted during ZIKV infection

**Fig 4:**
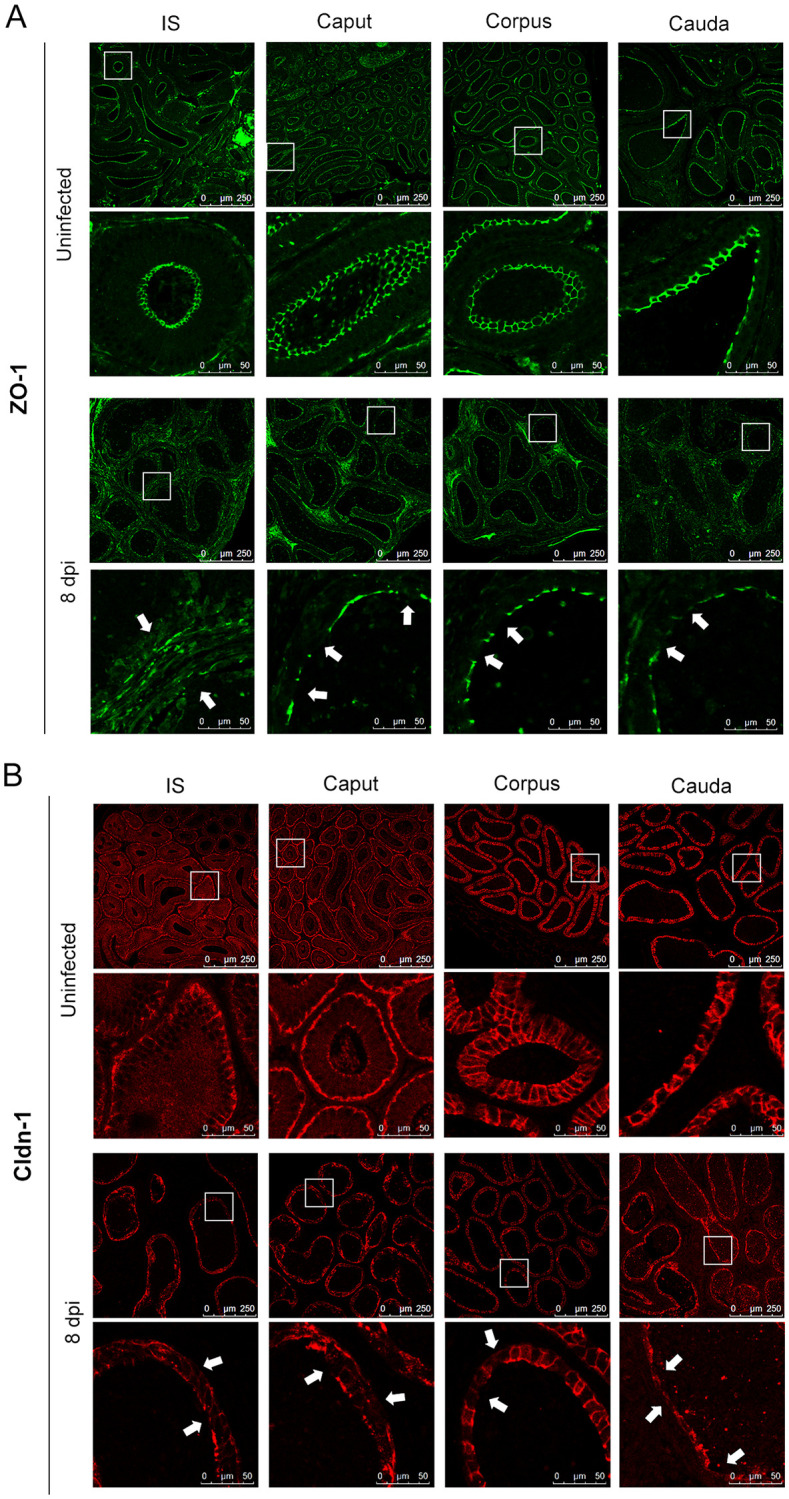
Expression and distribution of tight junction proteins in the epididymis after ZIKV infection. Eight-week-old male AG6 mice were inoculated with 10^5^ plaque-forming units (PFU) of ZIKV in bilateral footpads. n = 5. Epididymis was harvested at 8 days post infection (dpi), paraffin sections were prepared. ZO-1 (A) and Cldn-1 (B) in each segment were detected by immunofluorescent staining. Uninfected mice were used as a control. The disruptions were indicated by white arrows.

ZO-1 and Cldn-1 were detected to evaluate the BEB integrity after ZIKV infection. In the uninfected control, ZO-1 was located at the apical end and basal membranes of epithelial cells and in the area between the cells. A grid pattern was observed in all segments, and the expression was increased downstream along the tubule. Changes were not observed at 2 and 5 dpi (S2 Fig). However, at 8 dpi, ZO-1 was significantly disordered, expression levels were decreased, and grid arrangement disappeared. The continuity of ZO-1 was severely damaged in all segments (Fig 4A). Distribution of Cldn-1 was more complex than that of ZO-1. In the uninfected control, Cldn-1 was predominantly located in the basolateral membranes and the apical end of epithelial cells. Downstream, expression of Cldn-1 between the cells in the apical end was significantly increased forming a reticulate structure. However, at 5 dpi, ZIKV infection considerably reduced Cldn-1 expression and disrupted the arrangement. These changes were especially pronounced in the caput and cauda at 8 dpi, where regularly arranged structure of Cldn-1 was completely destroyed (Fig 4B). These results indicate that ZIKV destroyed the BEB by disrupting the expression and arrangement of ZO-1 and Cldn-1, which may lead to subsequent inflammatory cell infiltration.

### AQP1 and Lcn8 are downregulated by ZIKV infection

**Fig 5:**
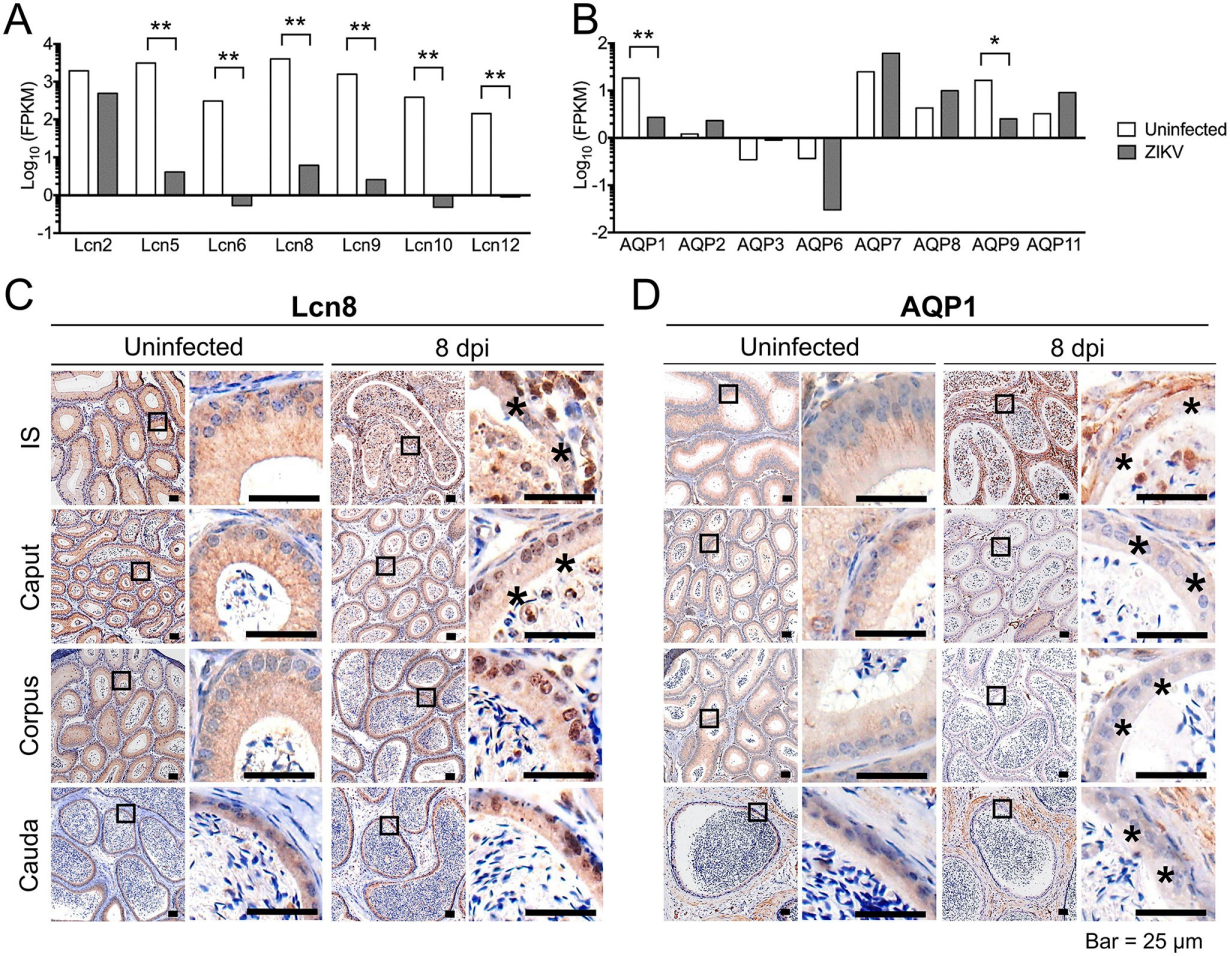
Changes in lipocalins (Lcns) and aquaporins (AQPs) in the epididymis after ZIKV infection. Eight-week-old male AG6 mice were inoculated with 10^5^ plaque-forming units (PFU) of ZIKV in bilateral footpads. Epididymis at 5 days post infection (dpi) was harvested and subjected to transcriptome analysis. Uninfected mice were used as the control. Transcription levels of selected genes associated with Lcns (A) and AQPs (B) were compared in ZIKV-infected and uninfected mice. FPKM referred to fragments per kilobase million. The results were expressed as Log_10_(FPKM). The results were pooled data from 3 mice. The significant differences were displayed according to q value, FDR-adjusted p-value of the test statistic. *, Q < 0.05, **, Q < 0.01. The distribution of Lcn8 (C) and AQP1 (D) in the epididymis at 8 dpi was analyzed by immunohistochemistry staining. The changes were indicated by asterisks.

Epididymal samples of ZIKV-infected mice at 5 dpi and uninfected mice were subjected to transcriptome analysis. The results indicated that ZIKV infection down-regulated the expression levels of Lcns and AQPs (Fig 5A and 5B). In the uninfected control, Lcn8 was distributed in the nuclei and cytoplasm of EEC especially in the apical border (Fig 5C). At 8 dpi, distribution of Lcn8 was significantly altered in the proximal end, especially in IS. An increase in the scattered antigen expression was observed in the lumen and interstitial tissue; however, the expression of Lcn8 in the epithelium was decreased. There were no obvious changes in the expression level and distribution of Lcn8 in downstream segments. These results suggested that ZIKV infection might reduce epididymal secretion mainly in the IS region (Fig 5C).

AQPs are essential regulators of the fluid movement. The expression of AQP1 is associated with the removal of water from the tubular spaces and facilitates sperm movement along the epididymis by maintaining pressure difference between the proximal and distal ends of the epididymis. Normally, AQP1 was mainly detected in the apical cytoplasm of EEC, and the density was gradually decreased downstream. After ZIKV infection, the expression of AQP1 in epithelial cells in all segments was downregulated. However, AQP1 was expressed in the interstitial tissue, especially in the cauda (Fig 5D). These data indicated that the resorption functionality were disrupted by ZIKV, which may be related to the enlargement of the lumen.

## Discussion

Numerous experimental studies revealed that ZIKV infection damaged testis and led to male infertility in mice [5–8]. The epididymis is an important organ of the male reproductive system that plays a role in transportation, maturation, and storage of the sperm. However, the effect of ZIKV infection on the epididymis was less investigated. In this study, we demonstrated that the epididymis was susceptible to ZIKV, and the infection induced epididymal injury according to the data of histomorphological examination and altered epididymal functionality in AG6 mice indicating that ZIKV infection might have additional important impacts on male reproductive health.

To investigate the characteristics of ZIKV infection in the epididymis, the kinetics of ZIKV replication and dissemination *in situ* were determined. The epididymis efficiently supported ZIKV replication (Fig 1A). Viral antigens were distributed in the interstitium around epididymal tubules on 5 dpi, but were yet observed in the lumen, suggesting that the viral antigen originated from the blood. Interestingly, at 8 dpi, high levels of the antigen with strong positive staining were mainly present in the lumen of IS (Fig 1B) suggesting that the viral antigen might originate from the virus replicating in the testis. At 8 dpi, EECs with strongly positive staining were detected from the caput to cauda indicating that EECs, especially principal cells, which constitute the majority of the epithelial layer, were susceptible to ZIKV (Fig 1B). Thus, it indicated that both hematogenous/lymphogenous and excurrent testicular routes contribute to the dissemination of ZIKV in the epididymis, and the former route is preceding. Epididymal lesions induced by ZIKV may start as an independent process associated with testicular infection. This result was confirming a previous report of Tsetsarkin et al. that demonstrated that hematogenous and excurrent testicular routes are involved in ZIKV spreading in the epididymis by using microRNA-targeted ZIKV clones [53]. Since the mouse model we used herein is immunodeficient, to verify the tropism of ZIKV, we further isolated primary EEC of the simian, which was immunocompetent host, and infected with ZIKV *in vitro*. SC and LC, which were proved as the targets of ZIKV, were also infected. The results confirmed EEC were susceptible to ZIKV. However, the distribution of viral antigens in EEC were different for that in SC or LC, which indicated ZIKV infection in epididymis might be complicated (Fig 1C and 1D). These indicated the susceptibility of epdidymis to ZIKV is more general.

Subsequently, the histomorphological changes in the epididymis after ZIKV infection were analyzed; the results indicated that epithelial structure was disrupted, especially at the proximal end (Fig 2A). At 5 dpi, considerable cell debris was detected in the proximal lumen suggesting impaired transport function. At 8 dpi, additional cell debris was detected in the lumen of the proximal end in combination with thinning of the epithelial layer and an increase in the luminal diameter indicating that epididymal damage might be aggravated by testicular infection (Fig 2B and 2C). According to TEM, significant histopathological changes were detected in the proximal end (the caput) and the distal end (the cauda) at 8 dpi, but more severe in the former. In the caput, the vesicles disappeared, and enlarged intercellular space in EEC indicated abnormal secretion and degradation of the physical barrier. In the cauda, where mature sperm is stored, sperm traversed the BEB and contacted with the basement membrane (Fig 2D). These changes suggested that ZIKV infection destroyed the epididymal structure required for sperm maturation and transport.

Generally, the inflammatory response is involved in the pathogenesis of acute infection diseases. The spectrum of cytokines was initially analyzed. At 8 dpi, the levels of IL-6 and IL-28 were increased compared to those of other cytokines tested in the epididymis; however cytokine levels were not significantly altered in the testis at the same time point (Fig 3A and 3B and S1 Fig). IL-6 is a lymphokine produced by activated T cells and fibroblasts and has various biological functions in immunity, tissue regeneration, and metabolism [54]. In this study, IL-6 production may contribute to the host defense against ZIKV infection. Excessive synthesis of IL-6 might be closely associated with ZIKV-induced epididymal lesions in AG6 mice. IL-28 is mainly expressed in activated mononuclear cells and dendritic cells [55]. Considerable increase in IL-28 may play an important antiviral role in ZIKV infection, and IL-28 may be used as an antiviral agent in ZIKV infection in the future. However, except for IL-6 and IL-28, no significant changes in the cytokine expression were detected apparently, possibly due to the immunoprivileged characteristics of the testis and epididymis or to a mouse model with knockout of IFN receptors used in this study.

Then, the effect of immunocytes on epididymal lesions was investigated by IHC using antibodies against CD45 (a leukocyte common antigen), CD11c (expressed at a high level in DCs), or F4/80 (macrophage marker). Considerable changes, including the presence of numerous triple marker-positive cells in the lumen in the caput and cauda at 8 dpi (Fig 3C), indicating significant infiltration of multiple types of inflammatory cells and possible consequential damage to the sperms during maturation and transport in the epididymis supporting our TEM observations (Fig 2D). Overall, our results suggest that excessive inflammatory response might also contribute to epididymal lesions.

The tight junctions were detected to evaluate the BEB. In this study, the expression and distribution of ZO-1 and Cldn-1 were analyzed to evaluate the effect of ZIKV on the BEB. After the infection, reduced expression and disordered distribution of ZO-1 and Cldn-1 were detected from IS to the cauda suggesting that the integrity of the BEB is severely disrupted in entire epididymis by ZIKV (Fig 4A and 4B). This result was confirmed by the leakage of sperm detected by TEM (Fig 2D). Since the sperm is immunogenic, intact BEB serves as a physical and immunological barrier and is vitally important to prevent sperm antigens from escaping the duct and contacting immune cells. ZIKV-induced BEB disruption, inflammatory reaction, and sperm leakage in the epididymis suggest possible immune damage of the sperm, which is closely associated with male reproductive health.

In addition to physical barrier functions, secretion and absorption are two other key functions responsible for the maintenance of the microenvironment suitable for the sperm. Small vesicles in epithelial cells detected by TEM are involved in secretion (Fig 2D). Lcns are the ancient family of proteins with important biological functions conserved among species. The basic function of Lcns is binding of small hydrophobic ligands, and Lcns function as lipophilic ligand carriers. PGDS is a lipocalin and serves as a biochemical marker of sperm quality in human semen [56]. Lcn5 and Lcn8, the rodent epididymal Lcns, bind retinoic acid, which is an important regulatory factor in the male reproductive tract and facilitates maturation of the sperm [33]. In this study, ZIKV infection resulted in significant downregulation of the Lcn gene clusters at transcriptional level and altered the distribution pattern of Lcn8 protein, especially in the IS region (Fig 5A and 5C), which may be attributed to ZIKV-induced disruption of the epithelium. These features indicated the alterations of the epididymal secretion and the changes in the sperm microenvironment after ZIKV infection.

AQPs are the family of water-transporting proteins that includes integral membrane proteins that form pores in the membrane of the cells to increase water movement across the biological membranes. The efferent ductules and IS in the epididymis play an important role in water absorption and sperm concentration [19,25]. Thus, the expression and distribution of AQPs was assayed. After ZIKV infection, the levels of AQP1 and AQP9 were significantly decreased, and the levels of other AQPs were increased at the transcriptional levels (Fig 5B), which might result from the disruption of EEC. Since the different locations and functions of AQP members, their changes might vary. AQP1 was present in EEC. The data of IHC indicated that AQP1 distribution in epithelial cells was changed to interstitial distribution after ZIKV infection, especially in IS (Fig 5D). The changes in the distribution pattern indicated that epididymal water adsorption might be impaired. Moreover, a substantial decrease in epithelial thickness and expansion of the lumen at the proximal ends suggest a dysfunctional regulation of water secretion and adsorption in the epididymal duct. Overall, the results suggested a disorder of secretion/absorption in the epididymis after ZIKV infection.

Totally, this study demonstrated that the epididymis was an important target of ZIKV in AG6 mice. ZIKV infected EEC, causing both anatomical and physiological changes. In addition, inflammatory infiltration was also observed. These might have a substantial influence on male reproductive health in mice. The study evaluates the pathogenesis in the epididymis after ZIKV infection by assessing part of the early immune response and the changes in epididymal functionality to reveal the potential effect of ZIKV to epididymis. Hence, ZIKV-induced lesions in the epididymis in humans cannot be ignored, and longitudinal studies in ZIKV-infected population in epidemic areas should be carried out.

## Supporting information

S1 Fig Cytokines in the epididymis and testis after ZIKV infection. Eight-week-old male AG6 mice were inoculated with 10^5^ PFU of ZIKV in bilateral footpads. Testis and epididymis were harvested at 2, 5, and 8 days post infection (dpi). Cytokine levels were determined by protein microarray. Uninfected mice were used as a control. The levels of the cytokines are shown as signal intensity. The results of 3 mice were pooled at each time point. 0 dpi refers to uninfected mice. *, P < 0.05 **, P < 0.01, according to the t test.
(TIF)

S2 Fig Expression and distribution of tight junction proteins in the epididymis at day 2 and 5 after ZIKV infection. Eight-week-old male AG6 mice were inoculated with 10^5^ plaque-forming units (PFU) of ZIKV in bilateral footpads. n = 5. Epididymis was harvested at 2, 5 days post infection (dpi), paraffin sections were prepared. ZO-1 (A) and Cldn-1 (B) in each segment were detected by immunofluorescent staining. Uninfected mice were used as a control. The histo-structure in ZIKV-infected epididymis at 2 and 5 dpi showed no evident change to the uninfected.
(TIF)
