# Supplementary figures and images for: Zika virus disrupts the barrier structure and Absorption/Secretion functions of the epididymis in mice
Source: PLoS Negl Trop Dis. 2021 Mar 5;15(3):e0009211. doi: 10.1371/journal.pntd.0009211 (PMC7968736; doi:10.1371/journal.pntd.0009211)

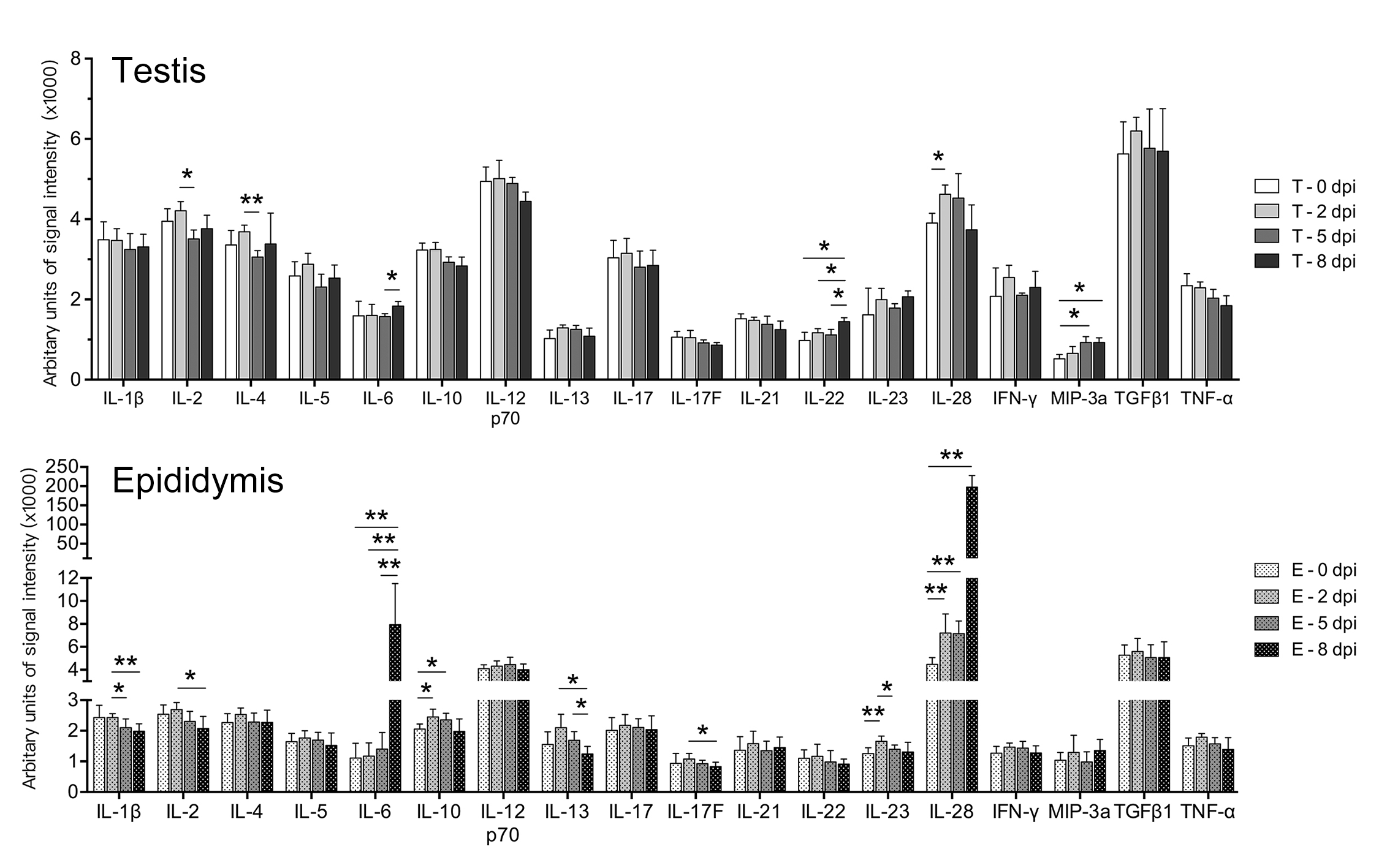

Supplement: S1 Fig — Eight-week-old male AG6 mice were inoculated with 105 PFU of ZIKV in bilateral footpads. Testis and epididymis were harvested at 2, 5, and 8 days post infection (dpi). Cytokine levels were determined by protein microarray. Uninfected mice were used as a control. The levels of the cytokines are shown as signal intensity. The results of 3 mice were pooled at each time point. 0 dpi refers to uninfected mice. *, P < 0.05 **, P < 0.01, according to the t test. (TIF) [file pntd.0009211.s001.tif]

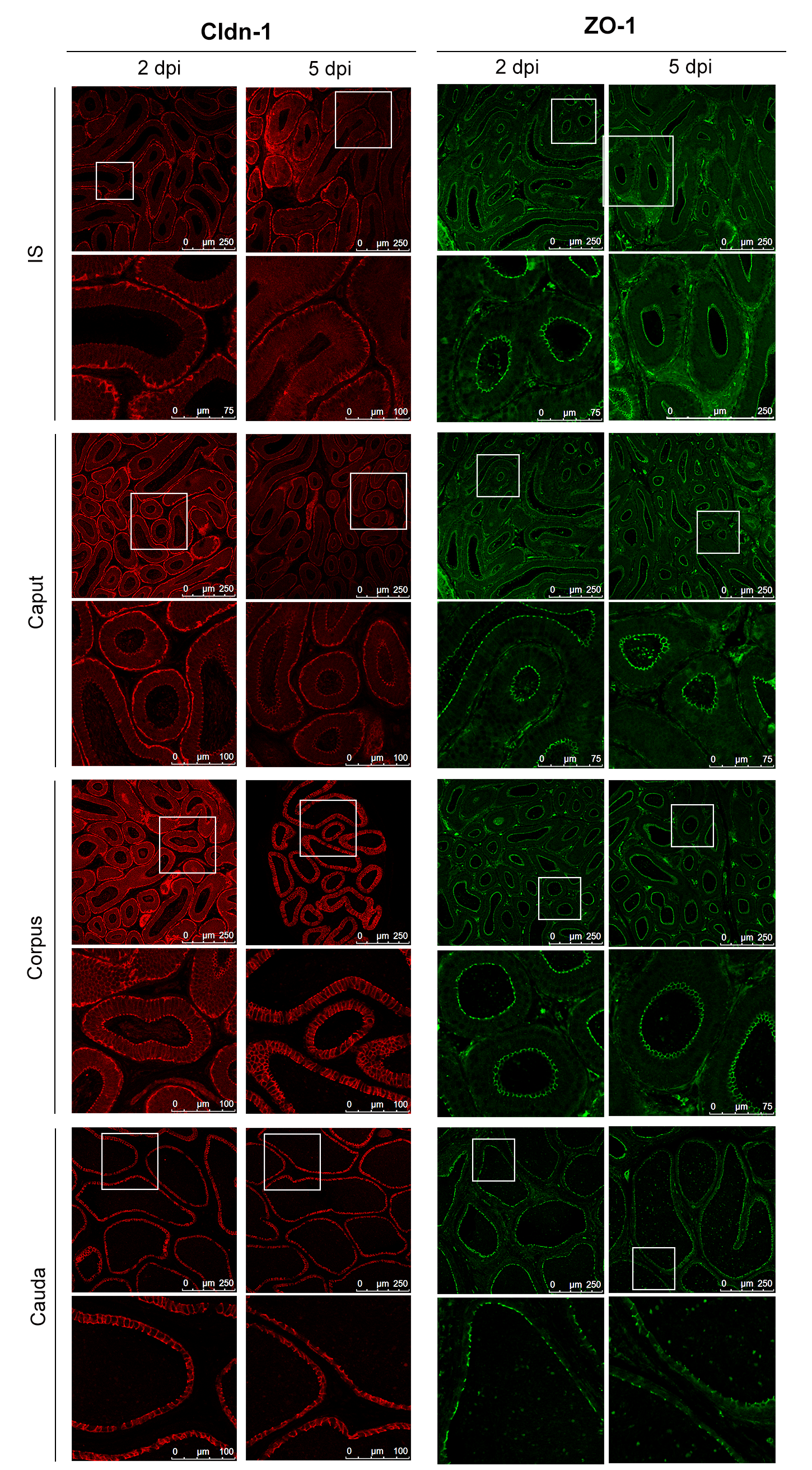

Supplement: S2 Fig — Eight-week-old male AG6 mice were inoculated with 105 plaque-forming units (PFU) of ZIKV in bilateral footpads. n = 5. Epididymis was harvested at 2, 5 days post infection (dpi), paraffin sections were prepared. ZO-1 (A) and Cldn-1 (B) in each segment were detected by immunofluorescent staining. Uninfected mice were used as a control. The histo-structure in ZIKV-infected epididymis at 2 and 5 dpi showed no evident change to the uninfected. (TIF) [file pntd.0009211.s002.tif]
